# Diaphragmatic ultrasound findings correlate with dyspnea, exercise tolerance, health-related quality of life and lung function in patients with fibrotic interstitial lung disease

**DOI:** 10.1186/s12890-019-0936-1

**Published:** 2019-10-21

**Authors:** Pauliane Vieira Santana, Leticia Zumpano Cardenas, André Luis Pereira de Albuquerque, Carlos Roberto Ribeiro de Carvalho, Pedro Caruso

**Affiliations:** 1 Pulmonary Division, Heart Institute (InCor), Hospital das Clínicas, Faculdade de Medicina da Universidade de São Paulo, São Paulo, Brazil; 2 Intensive Care Unit, AC Camargo Cancer Center, São Paulo, Brazil; 3 Hospital Sírio Libanês, São Paulo, Brazil

**Keywords:** Diaphragm, Ultrasonography, Interstitial lung diseases, Diagnostic imaging, Dyspnea, Exercise tolerance, Quality of life

## Abstract

**Background:**

Fibrotic interstitial lung disease (FILD) patients are typically dyspneic and exercise-intolerant with consequent impairment of health-related quality of life (HRQoL). Respiratory muscle dysfunction is among the underlying mechanisms of dyspnea and exercise intolerance in FILD but may be difficult to diagnose. Using ultrasound, we compared diaphragmatic mobility and thickening in FILD cases and healthy controls and correlated these findings with dyspnea, exercise tolerance, HRQoL and lung function.

**Methods:**

We measured diaphragmatic mobility and thickness during quiet (QB) and deep breathing (DB) and calculated thickening fraction (TF) in 30 FILD cases and 30 healthy controls. We correlated FILD cases’ diaphragmatic findings with dyspnea, exercise tolerance (six-minute walk test), lung function and HRQoL (St. George’s Respiratory Questionnaire).

**Results:**

Diaphragmatic mobility was similar between groups during QB but was lower in FILD cases during DB when compared to healthy controls (3.99 cm vs 7.02 cm; *p* <  0.01). FILD cases showed higher diaphragm thickness during QB but TF was lower in FILD when compared to healthy controls (70% vs 188%, *p* <  0.01). During DB, diaphragmatic mobility and thickness correlated with lung function, exercise tolerance and HRQoL, but inversely correlated with dyspnea. Most FILD cases (70%) presented reduced TF, and these patients had higher dyspnea and exercise desaturation, lower HRQoL and lung function.

**Conclusion:**

Compared to healthy controls, FILD cases present with lower diaphragmatic mobility and thickening during DB that correlate to increased dyspnea, decreased exercise tolerance, worse HRQoL and worse lung function. FILD cases with reduced diaphragmatic thickening are more dyspneic and exercise-intolerant, have lower HRQoL and lung function.

## Background

Dyspnea and exercise intolerance characterize fibrotic interstitial lung diseases (FILD) [1, 2]. Both are determinants of impaired health-related quality of life (HRQoL) observed in FILD patients [1, 3, 4]. Dyspnea and exercise intolerance in FILD have several underlying mechanisms [5]. Among them is respiratory muscle dysfunction [6, 7], which is usually difficult to diagnose [6].

In ILD, increased lung elastic recoil overloads the respiratory muscles [5, 8]. In addition, many other factors present in ILD, such as chronic hypoxemia, corticosteroids, systemic inflammation, physical inactivity and malnutrition may promote muscle dysfunction [6]. However, the few studies that have evaluated diaphragmatic function in ILD report conflicting results. While some authors have found preserved inspiratory muscle strength in ILD [5, 8–10], Walterspachen et al. [7] found reduced diaphragmatic strength, assessed with non-volitional measures, in a subset of ILD subjects with severe disease.

The diaphragm is the main inspiratory muscle being responsible for 60 to 70% of the tidal volume during quiet breathing [11, 12]. Due to its location and size, its function can be assessed by ultrasound (US). Initially, standardized techniques for mobility and diaphragmatic thickening measurements were reported in healthy volunteers [13–17]. Later, thoracic ultrasound (TUS) focusing on the diaphragm was employed in patients with respiratory disorders such as asthma [18], chronic obstructive pulmonary disease (COPD) [19], cystic fibrosis [20] and respiratory failure [21–23]. Recently, TUS was applied in FILD patients [24] and results have shown decreased diaphragmatic mobility during deep breathing that is associated with reduced lung volumes [24].

Improvement of HRQoL, including relief of dyspnea and exercise intolerance, is the cornerstone of the care of FILD patients, most of whom have irreversible lung diseases [25, 26]. In cases where TUS findings were correlated with dyspnea, exercise tolerance, HRQoL and lung function, US could be used to propose and monitor interventions such as rehabilitation and to assess disease progression [27].

Firstly, we hypothesized that diaphragmatic mobility and thickness, assessed by TUS, would be associated with dyspnea, exercise tolerance, HRQoL and lung function in FILD cases. Secondly, we hypothesized that a proportion of FILD cases would present with reduced diaphragmatic thickening and they would have different clinical and functional characteristics from FILD cases without reduced thickening.

## Methods

### Study design and subjects

We performed a prospective, observational study, involving 30 FILD cases and 30 healthy controls consecutively recruited from February 2014 to February 2016. Our local research ethics committee (Comissão de Ética para Análise de Projetos de Pesquisa do Hospital das Clinicas da Faculdade de Medicina da Universidade de São Paulo - CAPPesq 0835/11) approved the study and all subjects signed an informed consent.

FILD cases were recruited from an outpatient interstitial lung disease (ILD) clinic at a tertiary care teaching hospital. Diagnosis of ILD was based on established criteria which involved clinical features, lung function, chest computed tomography, bronchoalveolar lavage and, eventually, lung biopsy. Fibrotic interstitial lung disease was defined as ILD patients with radiological evidence of fibrosis and physiologic impairment (restrictive pattern in pulmonary function tests – FVC < 80% of predicted). FILD cases had to be clinically stable and without change of therapeutic regimen during the last 3 months. Exclusion criteria were a concurrent disorder, such as COPD, active infection or neuromuscular disease. In addition, we excluded subjects with ILD associated with connective tissue disease that had any sign, symptom (muscle pain or fatigue) or laboratorial suggestion of muscle involvement like myositis or myopathy (indicated by abnormal muscle enzymes or the presence of antisynthetase antibody). None of the patients suffered from dermato-polymyositis. Healthy controls were at least 18 years old and without any cardiopulmonary or neuromuscular disease.

For all subjects, we recorded demographic data including smoking habits. From FILD cases, we recorded comorbidities, medications in use (eg. corticosteroids and immunosuppressants) and resting dyspnea, quantified by the Medical Research Council (MRC) scale [28]. Dyspnea was classified as mild (MRC = 1), moderate (MRC = 2 or 3) and severe (MRC = 4 or 5). All subjects underwent pulmonary function test (PFT) and TUS. Only FILD cases answered a HRQoL questionnaire and performed a six-minute walk test (6MWT).

PFT (Elite Dx, Medical Graphics Corporation, St. Paul, MN, USA) measured the forced vital capacity (FVC), forced expiratory volume during the first second (FEV_1_) and inspiratory capacity (IC). FILD cases also underwent functional residual capacity (FRC) and total lung capacity (TLC) measurements. Tests were performed according to ERS/ATS Statement [29] and predicted values were derived from a sample of the Brazilian population [30].

An Additional file 1: Methods and Results describes further details about the measurement of respiratory muscle strength, quality of life assessment (HRQoL) and exercise capacity evaluation (6MWT).

Maximal inspiratory pressure (MIP), maximal expiratory pressure (MEP) and sniff nasal inspiratory pressure (SNIP) were measured (Respiratory Pressure Meter, CareFusion, CA-USA) in all subjects according to standardized criteria [31]. For MIP and MEP, the highest value of three maneuvers was recorded for analysis. For SNIP, the highest value of ten efforts was used for analysis [31]. Predicted values were derived from a sample of the Brazilian population [32, 33].

The HRQoL was quantified using the St. George’s Respiratory Questionnaire (SGRQ), a respiratory-specific HRQoL questionnaire with three different domains: respiratory symptoms, activity and psychosocial impact of the disease. Higher scores (range from 0 to 100) correspond to worse HRQoL. Although developed for COPD, validity and reliability of the SGRQ in ILD patients has been determined [1, 34, 35].

The 6MWT was performed according to standardized criteria [36]. Before and after the test, heart rate, peripheral oxygen saturation and modified Borg scale [37] were measured. Predicted values were derived from a sample of the Brazilian population [38].

#### Thoracic ultrasound focused on diaphragm (TUS)

TUS was performed using a portable system (Nanomaxx, Sonosite, Bothell, WA, USA) or another machine (M-Turbo, Sonosite) with subjects in a semi-recumbent position. For diaphragmatic mobility evaluation, a convex transducer (2–5 MHz) was placed over the right anterior subcostal region between the midclavicular and anterior axillary lines. The transducer was directed medially, cephalad and dorsally, so that the US beam reached perpendicularly the posterior third of the right diaphragm. After obtaining the best view on two-dimensional mode, the mobility was measured through the M-mode, from the amplitude of cranio-caudal diaphragmatic excursion during quiet breathing (QB) and deep breathing (DB). To record the TUS at DB, all subjects were asked to perform a maximum inspiratory effort (maximum inspiratory capacity maneuver) for at least 2 s, to attain a maximal lung volume close to the total lung capacity (TLC) [14, 15, 17]. For diaphragmatic thickness evaluation, a linear transducer (6–13 MHz) was placed over the zone of apposition of the diaphragm to the rib cage, between the right anterior and medial axillary lines. Using the two-dimensional mode, the diaphragm was observed as a structure composed of a non-echogenic central bordered by two hyperechogenic (peritoneal and pleural) layers [13, 15, 16]. Diaphragmatic thickness was measured from the deepest pleural line to the deepest peritoneal line. First, we measured the thickness during QB at FRC (Tmin) and then, after a maximal DB, at TLC (Tmax). The diaphragm’s thickening fraction (TF) was calculated as TF = [(Tmax − Tmin)/Tmin] × 100.

One physician (PVS) with experience in TUS performed all measurements. At least three measurements of the diaphragm excursion and thickness were taken for all subjects and the average of the individual values was reported.

#### Statistical analysis

Statistical analyses were performed with SPSS software version 20.0 (IBM Corporation, Armonk, NY-USA). Data are presented as mean ± standard deviation or median and 25–75% interquartile range, as appropriate. Categorical data are presented as absolute and relative frequency. Student’s T-test was used to compare the normally distributed data (means) and Mann–Whitney test was used to compare data that were not normally distributed (medians). Categorical variables were compared using the chi-squared test or Fisher’s exact test. Correlations were analyzed using Spearman’s coefficient. Reduced diaphragmatic thickening was defined by diaphragmatic thickening fraction values below the 95% confidence interval of values obtained from the healthy controls. *P* values < 0.05 were considered statistically significant. We calculated the sample size based on data from a previous study by our group that examined diaphragmatic mobility and thickness in FILD patients. Considering a two-sided type I error of 0.05, a type II error of 0.20 and an expected difference of thickness at total lung capacity of 0.8 cm, at least 25 subjects per group were estimated to compare TUS findings between FILD cases and healthy controls.

## Results

Demographic and clinical data of FILD cases and healthy controls are depicted in Table 1. The diaphragmatic mobility and thickness were measured for all subjects. The classification of FILD patients and the current use of steroids are presented in Table 1. Two patients were on long-term oxygen therapy. Respiratory muscle strength was similar between FILD cases and healthy controls (Table 1). The prevalence of comorbidities was similar between FILD cases and healthy controls (Additional file 1: Methods and Results). The scores of SGRQ revealed a reduction in HRQoL in all domains (Additional file 1: Table SA1, Results) in FILD cases when compared to reference values [39].

**Table 1:** Characteristics of healthy controls and FILD cases

Variables	Controls (*n* = 30)	FILD (*n* = 30)	*p*
Age (years)	47 ± 16	49 ± 17	0.62
Male (%)	12 (40)	18 (60)	0.19
Body mass index (kg/m^2^)	27 ± 9	26 ± 4	0.47
Smoking status			0.24
Never (%)	23 (76.6)	24 (80)	
Past (%)	7 (23.4)	4 (13.3)	
Current (%)	0	2 (6.7)	
FVC (% predicted)	93 ± 13	58 ± 16	< 0.01
FEV_1_ (%predicted)	92 ± 12	62 ± 17	< 0.01
FEV_1_/FVC ratio	1.07 ± 0.20	0.86 ± 0.06	< 0.01
TLC (%predicted)	–	64 ± 14	
DL_CO_ (%predicted)	–	43 ± 15	
ILD diagnoses			
FHP		11	
ILD associated with CTD		7	
Non-classified IIP		5	
Idiopathic NSIP - fibrosing pattern		4	
IPF		2	
Sarcoidosis with fibrotic pattern		1	
Corticosteroid use	–		
Never, n (%)	–	17 (56.6)	
Current, Prednisone < 20 mg/d n (%)	–	8 (26.6)	
Current, Prednisone ≥ 20 mg/d	–	5 (16.6)	
Resting dyspnea (MRC) (%)			
1	–	5 (16.7)	
2	–	11 (36.7)	
3	–	9 (30.0)	
4	–	4 (13.3)	
5	–	1 (3.3)	
MIP (cmH_2_0)	95 ± 34	97 ± 34	0.86
MIP (%predicted)	89 ± 22	81 ± 24	0.20
MEP (cmH_2_0)	98 ± 32	94 ± 36	0.58
MEP (%predicted)	93 ± 32	87 ± 28	0.44
SNIP (cmH_2_0)	91 ± 23	92 ± 23	0.82
SNIP (%predicted)	81 ± 20	85 ± 24	0.45

Data expressed as mean ± SD

*FILD* fibrotic interstitial lung disease, *BMI* body mass index in kg/m^2^, *FVC* forced vital capacity, *FEV*_*1*_ forced expiratory volume in 1 s, *TLC* total lung capacity, *DL*_*CO*_ carbon monoxide diffusing capacity, *FHP* fibrotic hypersensitivity pneumonitis, *ILD* associated with *CTD* interstitial lung disease associated with connective tissue disease, *IIP* idiopathic interstitial pneumonia, *NSIP* non-specific interstitial pneumonia, *IPF* idiopathic pulmonary fibrosis, *mg/d* milligrams per day, *MRC* Medical Research Council, *MIP* maximal inspiratory pressure, *MEP* maximal expiratory pressure, *SNIP* sniff nasal inspiratory pressure

FILD cases walked less than predicted and presented peripheral oxygen desaturation, increased heart rate, dyspnea and leg fatigue at the end of the 6MWT (Additional file 1: Table SA2).

Diaphragmatic mobility during QB was similar between FILD and control groups (*p* = 0.95). However, during DB, diaphragmatic mobility was lower in the FILD cases when compared to healthy controls (*p* <  0.01). During QB, at FRC, the diaphragm of FILD cases was significantly thicker than the healthy controls (*p* = 0.01). But, after a maximal DB, at TLC, the diaphragm of FILD cases was significantly thinner than the healthy controls (*p* <  0.01), resulting in a lower TF in the FILD cases (*p* <  0.01). (Table 2 and Additional file 2: Figure S1).

**Table 2:** Diaphragmatic mobility, thickness and thickening fraction in FILD cases and healthy controls

Variables	Healthy controls (*n* = 30)	FILD cases (*n* = 30)	*p*
	Diaphragmatic mobility		
Quiet breathing - (cm)	1.54 (1.16–1.82)	1.41 (1.15–2.16)	0.95
Deep breathing - (cm)	7.02 (5.76–7.73)	3.99 (3.23–5.68)	< 0.01
	Diaphragmatic thickness and thickening fraction		
At FRC (rest, QB) (cm)	0.17 (0.15–0.20)	0.20 (0.17–0.23)	0.01
At TLC (DB) (cm)	0.54 (0.42–0.60)	0.34 (0.26–0.45)	< 0.01
Thickening fraction (%)	188 (148–239)	70 (49–108)	< 0.01

Data expressed as median (25th–75th interquartile range)

*FILD* fibrotic interstitial lung disease, *FRC* functional residual capacity, *TLC* total lung capacity

During DB, diaphragmatic mobility and thickness correlated with lung function (FVC, FEV_1,_ TLC and DLCO), exercise tolerance and HRQoL but negatively correlated with resting dyspnea. During DB, lesser diaphragmatic mobility and thickness correlated with more resting dyspnea, more desaturation and dyspnea at the end of the 6MWT; quality of life is worse (mainly respiratory symptoms and activity domain of SGRQ). (Table 3 and Additional file 3: Figure S2 and Additional file 4: Figure S3). However, nor corticosteroid use, nor a specific group of FILD diagnoses were associated with diaphragmatic mobility and thickness in FILD cases.

**Table 3:** Correlations between diaphragmatic ultrasound findings with resting dyspnea, exercise tolerance, quality of life and pulmonary function in FILD cases

Variables	Deep breathing mobility		Deep breathing thickness		Thickening fraction	
	*R*	*p*	*R*	*p*	*R*	*p*
	Health-related quality of life -SGRQ					
Respiratory symptoms	− 0.40	0.03	− 0.46	0.01	− 0.26	0.16
Activity	−0.45	0.01	−0.50	< 0.01	−0.44	0.01
	Resting dyspnea					
MRC scale	−0.57	< 0.01	− 0.36	0.05	− 0.54	< 0.01
	Pulmonary function test					
FVC (% pred)	0.76	< 0.01	0.70	< 0.01	0.68	< 0.01
DL_CO_ (% pred)	0.59	< 0.01	0.53	0.01	0.45	0.03
	Exercise tolerance					
SpO_2_ desaturation at end 6MWT	−0.41	0.03	−0.26	0.18	− 0.41	0.03
Borg dyspnea at end 6MWT	−0.41	0.03	−0.37	0.05	−0.50	< 0.01

Desaturation = (initial peripheral arterial oxygen saturation minus final saturation) over peripheral arterial oxygen saturation initial saturation, in percentage

*FILD* fibrotic interstitial lung disease, *MRC* Medical Research Council, *FVC* forced vital capacity, *FEV*_*1*_ forced expiratory volume in 1 s, *TLC* total lung capacity, *DL*_*CO*_ carbon monoxide diffusing capacity, *SpO2* peripheral oxygen saturation, *6MWT* six-minute walk test

For the healthy controls, the 95% confidence interval for TF during DB was 101 to 354%. To define the FILD cases with reduced diaphragmatic thickening, the choice of TF < 101% represents the values below which only 5% of the healthy controls’ values fall (5th percentile). Seventy percent of FILD cases presented reduced diaphragmatic thickening (Table 4). FILD cases with reduced diaphragmatic thickening had lower lung volumes (FVC and FEV_1_), higher resting dyspnea, worse HRQoL (activity and total domains of SGRQ), higher desaturation and dyspnea after the 6MWT (Table 4). Age, sex, BMI and corticosteroid were similar among FILD cases with and without reduced diaphragmatic thickening.

**Table 4:** Clinical, functional, exercise tolerance and HRQoL in FILD cases with and without reduced diaphragmatic thickening

Variable	Non-reduced diaphragmatic thickening (*n* = 9)	Reduced diaphragmatic thickening (*n* = 21)	*p*
Age	54 ± 14^a^	47 ± 16^a^	0.22
Sex, male (%)	5 (55.6%)	13 (61.9)	0.52
Body mass index (Kg/m^2^)	27 ± 3^a^	26 ± 4^a^	0.42
Lung function			
FVC (% predicted)	69 ± 12^a^	53 ± 15^a^	< 0.01
DLCO (%predicted)	48 ± 12^a^	40 ± 16^a^	0.25
MIP (% predicted)	90 ± 22^a^	77 ± 25^a^	0.17
MEP (% predicted)	95 ± 27^a^	83 ± 28^a^	0.30
SNIP (% predicted)	89 ± 5^a^	87 ± 12^a^	0.66
MRC dyspnea scale	2 (1–2.5)^b^	3 (2–3.5)^b^	0.01
Health-related quality of life			
Respiratory symptoms	29.6 (7.7–52.2)^b^	42.0 (25.0–57.0)^b^	0.17
Activity	37.8 (29.4–60.9)^b^	66.3 (38.6–76.3)^b^	0.03
Six-minute walk test			
Walked distance (m)	516 (405–576)^b^	499 (435–552)^b^	0.89
Walked distance (%predicted)	93 (84–103)^b^	83 (77–94)^b^	0.25
Initial SpO_2_	96 (93–96)^b^	95 (93–96)^b^	0.76
Final SpO_2_	91 (83–94)^b^	85 (68–90)^b^	0.19
SpO_2_ desaturation at end 6MWT 6MWT	5 (2–10)^b^	11 (5–25)^b^	0.04
Initial heart rate (ppm)	80 (69–91)^b^	84 (70–98)^b^	0.80
Final heart rate (ppm)	104 (88–141)^b^	119.5 (107–136)	0.31
Initial Borg dyspnea scale	0 (0–1)^b^	0 (0–0.3)^b^	0.89
Final Borg dyspnea scale	5 (3–5)^b^	7 (5–8)^b^	0.04
Initial Borg leg fatigue scale	0^b^	0^b^	0.93
Final Borg leg fatigue scale	3 (1–6)^b^	5 (1.5–7)^b^	0.28

Reduced diaphragmatic thickening was defined by diaphragmatic thickening fraction values below the 95% confidence interval of values obtained from the healthy controls”

*FVC* forced vital capacity, *FEV*_*1*_ forced expiratory volume in 1 s, *TLC* total lung capacity, *DL*_*CO*_ carbon monoxide diffusing capacity, *MRC* Medical Research Council, *SpO*_*2*_ peripheral capillary oxygen saturation, *Ppm* pulse per minute

^a^Data expressed as mean ± SD

^b^Data expressed as median (25th–75th interquartile range)

## Discussion

The novel findings of this study are that in FILD cases compared to healthy adults, lower deep breathing diaphragmatic mobility and thickening correlated with increased dyspnea, decreased exercise tolerance, worse HRQoL and worse lung function. Most FILD cases (75%) presented reduced diaphragmatic thickening and these patients had higher dyspnea, higher desaturation, worse HRQoL and lung function than FILD cases without reduced diaphragmatic thickening. In addition, FILD cases presented a thicker diaphragm at rest compared to healthy controls. Using US, we assessed the diaphragm function of a heterogeneous sample of FILD patients. Findings of higher dyspnea, exercise intolerance, worse HRQoL and lung function in FILD cases with reduced diaphragmatic mobility and thickening are novel and provide further evidence that diaphragm function affects FILD patients clinically.

Few studies explored the relationship between diaphragmatic function, dyspnea, HRQoL and exercise tolerance in FILD. In our study, FILD cases with lower DB diaphragmatic mobility and TF had more resting and exertional dyspnea and worse HRQoL. Further, the lower the DB diaphragmatic mobility and the TF, the greater the desaturation at the end of the 6MWT. The reduced diaphragmatic mobility and thickening in FILD cases may compromise the overloaded inspiratory muscles [40, 41] eliciting early onset breathlessness [5, 41] resulting in poor HRQoL [1, 3]. In addition, impairment in diaphragm function may hinder ventilation throughout exercise causing additional mismatch on the ventilation to perfusion ratio, leading to desaturation similar to that shown in diaphragm paralysis [42].

The relationship between muscle function, dyspnea and HRQoL has been explored in sarcoidosis [43, 44] lung cancer patients [45] and in COPD [46]. Interestingly, in COPD, Paulin et al. [46] used TUS to assess diaphragm function and showed that COPD patients with lower diaphragmatic mobility also had lower 6MWT performance and higher exertional dyspnea [46]. Furthermore, air trapping influenced the diaphragmatic mobility in COPD. The authors hypothesize that COPD patients with air trapping imposed a mechanical disadvantage of the inspiratory muscles due to their shortened lengths, which was accentuated during exertion and elicited breathlessness [46].

In our study, diaphragmatic mobility and TF was associated with dyspnea and desaturation, but did not influence the 6MWT distance covered by FILD cases.

To the best of our knowledge, only two studies used the TUS to investigate the diaphragmatic function in ILD. He et al. [47] showed that diaphragmatic mobility, but not thickness, was similar between a small sample of idiopathic pulmonary fibrosis patients and healthy controls. In contrast, we previously recorded that FILD patients presented reduced diaphragmatic mobility and TF during DB when compared to healthy controls [24]. Diaphragmatic mobility during DB was associated with FVC, FEV_1_ and TLC in FILD [24].

The present study reinforced those findings. We found a strong correlation between diaphragmatic mobility and TF with lung function. Although expected and intuitive, these correlations are not consistent findings even in healthy adults. While some studies found a linear relationship between diaphragmatic mobility and lung volumes in healthy volunteers [48, 49], another study showed that diaphragmatic mobility measured during QB and DB or inspiratory capacity maneuvers poorly correlated with lung volumes [50]. Recently, in 210 healthy volunteers, Boussuges et al. [14] found only a weak correlation between diaphragmatic mobility and lung volumes. This controversy may be explained by the fact that inspiratory lung volumes are determined by diaphragmatic mobility, and also by recruitment of extra diaphragmatic muscles [51] and thoracoabdominal compliance [14]. These factors also influence the thickening of diaphragm. In healthy subjects, thickening of diaphragm is greatest in the zone of apposition, during a primarily abdominal breath [52] and is affected by the diaphragm curvature [53].

In previous studies in COPD, both diaphragmatic mobility [54] and thickening [55] have been correlated with air trapping and airway obstruction. In our study, decreased diaphragmatic mobility seen in FILD patients during DB may reflect the reduced lung volume. The reduced diaphragmatic thickening may be due to an intrinsic muscle dysfunction associated with several causal factors (inflammatory and oxidative stress, corticosteroid use, physical inactivity and malnutrition) usually present in ILDs [6, 7]. We cannot rule out that the curvature of the diaphragm may have affected diaphragmatic thickening. The lower lung volumes in ILDs decrease the radius of the diaphragmatic curvature, which may explain our finding of increased diaphragmatic thickness in patients with FILD. Increased diaphragmatic thickness could also represent muscle hypertrophy consistent with a training effect, and has been showed in other respiratory diseases such as chronic asthma [18] and cystic fibrosis [20].

Respiratory muscle strength was similar between the two FILD subgroups (with and without reduced diaphragmatic thickening). Respiratory muscle function is often neglected in ILD and the few studies that have addressed it report conflicting results. Preserved inspiratory strength in ILD has been described [8–10], but recently reduced diaphragmatic strength in a sample of patients with more severe ILD was shown [7]. These conflicting results may reflect a “set of opposing forces” in ILD. ILD subjects have a mechanical advantage of inspiratory force generation at lower operating lung volumes and a “training effect” on the diaphragm because of the lung stiffness and increased elastic recoil [6]. However, several factors (hypoxemia, inflammatory status, corticosteroids, physical inactivity and malnutrition) may be harmful for the respiratory muscles in ILD [6]. Overall, the impaired diaphragmatic function (reduction of mobility and thickening) may represent the harmful effects on respiratory muscles, while “training effect” maintains preserved inspiratory strength in these ILD patients. Further, the heterogeneity and severity of the underlying ILD may be a confounding factor in the interpretation of respiratory muscle assessment.

In our study, corticosteroid was not associated with impaired diaphragmatic function in FILD cases possibly because only 5 patients (16,6%) were actually using a dosage of prednisone higher than 20 mg/day which is recognized as myotoxic. Corticosteroid use has been associated with mitochondrial dysfunction and oxidative stress leading to corticosteroid-induced myopathy [56]. However, the occurrence of corticosteroid-induced myopathy may be influenced by the dosage. Unfortunately, we did not investigate prospectively before and under steroid treatment for FILD. Our findings suggest that TUS may be used to monitor variables that are clinically relevant and prognostic determinants in FILD. In addition, identifying impairment of diaphragm function may alert physicians to avoid or minimize the use of myotoxic drugs, such as corticosteroids. Characterization of diaphragm function with TUS could further suggest targeted actions to improve function, such as rehabilitation, a recognized intervention to improve exercise tolerance and HRQoL and to decrease dyspnea.

Our study has some limitations. First, we studied a heterogeneous sample of FILD patients, which could be a confounding factor. However, heterogeneity is usual among FILD and gives a pragmatic character to the present study. Secondly, lung volumes were not measured concurrently with diaphragm thickness and mobility. Normalizing the mobility and thickness for lung volumes measured during the protocol could add to our understanding about the relationship between diaphragm function and lung volume restriction. Thirdly, TUS was assessed only on the right side. Fourthly, only one observer performed the TUS. However, we used M-mode to measure diaphragm mobility and standardized technique to evaluate diaphragm thickness, and both have shown to have a high reproducibility. A fifty consideration was that respiratory muscle strength was similar, which might be a reflection of a volitional measurement of overall respiratory muscle strength. Perhaps TUS discloses diaphragm function impairment before strength reduction, or the activity of global inspiratory muscle may compensate for diaphragmatic weakness. A sixth consideration was the performance of multiple comparisons in our study, which present the risk of erroneously finding a significant difference by chance. However, our study aimed to understand whether diaphragmatic ultrasound findings would correlate with clinically relevant parameters for ILD subjects, such as dyspnea and exercise intolerance that are determinants of their impaired quality of life (HRQoL). Lastly, our healthy controls did not have TLC measurements, but we have no reasons to believe that they could have any restriction or hyperinflation.

## Conclusions

Compared with healthy controls, FILD cases had higher diaphragmatic thickness at rest, but lower diaphragmatic mobility and thickening fraction during deep breathings that correlate to dyspnea, exercise tolerance, HRQoL and lung function. FILD cases with diaphragmatic reduced thickening have higher dyspnea, less exercise tolerance, lower HRQoL and lung function than FILD cases without reduced thickening.

Diaphragmatic US in FILD cases may be a useful tool to investigate and monitor diaphragm function, to propose and monitor interventions such as rehabilitation and use of pharmacological treatments such as corticosteroids.

## Supplementary information

**Additional file 1: **Additional file methods and results. This file contains information regarding to methodology and results. The subsection Additional file results present two tables. **Table SA1.** Health related quality of life according to St. George’s Respiratory Questionnaire in fibrotic interstitial lung disease (FILD) cases. **Table SA2.** Results of six-minute walk test in the fibrotic interstitial lung disease (FILD) cases.

**Additional file 2: **Line graph depicting diaphragmatic mobility at quiet (QB) and deep breathing (DB) and diaphragmatic thickness at functional (FRC) and total lung capacity (TLC) of all subjects (healthy controls and FILD cases). **Figure S1.** Comparison of diaphragmatic mobility between healthy controls and FILD cases. Diaphragmatic mobility during QB was similar between groups (*p* = 0.95), but during DB, diaphragmatic mobility was lower in the FILD cases when compared to healthy controls (*p* <  0.01). At FRC, the diaphragm of FILD cases was significantly thicker (p 0.01) than the healthy controls. But, at TLC, the diaphragm of FILD cases was significantly thinner than the healthy controls (*p* < 0.01).

**Additional file 3: Figure S2.** Correlation of diaphragmatic mobility during deep breathing and healthy-related quality of life (SGRQ), basal dyspnea (MRC), lung function (FVC) and exercise tolerance during the six-minute walk test among the fibrotic interstitial lung disease (FILD) patients.

**Additional file 4: Figure S3.** Correlation of diaphragmatic thickening fraction with quality of life (SGRQ), basal dyspnea (MRC), lung function (FVC) and exercise tolerance (desaturation and dyspnea at the end of the six-minute walk test) in patients with fibrotic interstitial lung disease (FILD) patients.

## Data Availability

The datasets used and/or analysed during the current study are available from the corresponding author on reasonable request.

## Abbreviations

6MWT: Six-minute walk test
ATS: American thoracic society
COPD: Chronic obstructive pulmonary disease
DB: Deep breathing
DL_CO_: Diffusing capacity of the lung for carbon monoxide
FEV1: Forced expiratory volume during the first second
FHP: Fibrotic hypersensitivity pneumonitis
FILD: Fibrotic interstitial lung diseases
FRC: Functional residual capacity
FVC: Forced vital capacity
HRQoL: Health-related quality of life
IC: Inspiratory capacity
ILD associated with CTD: Interstitial lung disease associated with connective tissue disease
MEP: Maximal expiratory pressure
MIP: Maximal inspiratory pressure
MRC: Medical Research Council
Non-classified IIP: Non-classified idiopathic interstitial pneumonia
NSIP with fibrosing pattern: Non-specific interstitial pneumonia with fibrosing pattern
QB: Quiet breathing
SGRQ: Saint George’s respiratory questionnaire
SNIP: Sniff nasal inspiratory pressure
SpO2: Peripheral oxygen saturation
TF: Thickening fraction
TLC: Total lung capacity
TUS: Thoracic ultrasound (TUS) focused on diaphragm
UIP: Usual interstitial pneumonia
US: Ultrasound
