# Supplementary material for: Diaphragmatic ultrasound findings correlate with dyspnea, exercise tolerance, health-related quality of life and lung function in patients with fibrotic interstitial lung disease
Source: BMC Pulm Med. 2019 Oct 21;19:183. doi: 10.1186/s12890-019-0936-1 (PMC6802109; doi:10.1186/s12890-019-0936-1)
Supplement: Supplementary file 1 — Additional file 1: Additional file methods and results. This file contains information regarding to methodology and results. The subsection Additional file results present two tables. Table SA1. Health related quality of life according to St. George’s Respiratory Questionnaire in fibrotic interstitial lung disease (FILD) cases. Table SA2. Results of six-minute walk test in the fibrotic interstitial lung disease (FILD) cases. [file 12890_2019_936_MOESM1_ESM.docx]

***ADDITIONAL FILES***

**Diaphragmatic ultrasound findings correlate with dyspnea, exercise tolerance, health-related quality of life and lung function in patients with fibrotic interstitial lung disease.**

Pauliane Vieira Santana, MD, PhD ^1,2^

Leticia Zumpano Cardenas, RT, PhD^1,2^

André Luis Pereira de Albuquerque, MD, PhD ^1,3^

Carlos Roberto Ribeiro de Carvalho, MD, PhD ^1^

Pedro Caruso, MD, PhD ^1,2^

^1^ Pulmonary Division, Heart Institute (InCor), Hospital das Clínicas, Faculdade de Medicina da Universidade de São Paulo, São Paulo, Brazil

^2^ Intensive Care Unit, AC Camargo Cancer Center, São Paulo, Brazil

^3^ Hospital Sírio Libanês, São Paulo, Brazil

***- Additional files – Methods and Results***

***Additional files - Methods***

***Inspiratory and expiratory muscle strength measurement.***

Maximal inspiratory pressure (MIP), maximal expiratory pressure (MEP) and sniff nasal inspiratory pressure (SNIP) were measured in FILD cases and healthy controls in a sitting position (Respiratory Pressure Meter, CareFusion, CA-USA).

During the MIP measurements, subjects were asked to exhale to residual volume and then perform a maximal inspiratory effort against an occluded mouthpiece, sustaining it for 3 seconds. To prevent artefactual measurements of airway opening pressure (Pao) due to buccal muscle and glottal closure we used a 2-mm wide leak at one end of the mouthpiece. The MEP measurements were measured from total lung capacity. The subjects were instructed to forcefully exhale against an occluded mouthpiece for 3 seconds or more. For MIP and MEP, subjects performed three maneuvers (or more since the last value was not higher than 20% from the previous), and the highest value was recorded for analysis. Predicted values were derived for the Brazilian population. During the SNIP measurement, one nostril was completely occluded by a nose plug connected to a pressure transducer. After a period of quiet breathing, subjects were asked to perform a strong, sharp, maximal sniff (the maneuver should be short than 500 ms and explosive so that it causes the collapse of the unplugged nostril). For SNIP, a minimum of ten efforts were made with rest intervals of 30 seconds and the peak pressure was recorded. The highest pressure recorded was used for analysis.

***Health-related quality of life (HRQoL)***

The HRQoL was quantified using the St. George's Respiratory Questionnaire (SGRQ), which is a respiratory-specific HRQoL questionnaire composed of 76 questions with three different domains: respiratory symptoms, activity and psychosocial impact of the disease. The scores range from 0 to 100 for all them and higher scores correspond to worse HRQoL. Although developed for chronic obstructive pulmonary disease, the validity and reliability of the SGRQ were already evaluated in interstitial lung disease patients.

***The six-minute walk test (6MWT)***

The 6MWT was performed according to standardized criteria. FILD cases remained sat for at least 10 minutes before the 6MWD test. Before and after the test, heart rate, peripheral oxygen saturation and modified Borg scale were measured. FILD cases were instructed to walk as far as possible along a 30-meter corridor for six minutes, to achieve their maximum possible walking distance. Standardized instructions were provided at each minute. FILD cases could stop and rest if desired but were encouraged to restart as soon as possible. Predicted values were derived for the Brazilian population.

***Additional files - Results***

Regarding to comorbidities, among FILD cases, four patients had hypertension, one patient had hypertension and hyperlipidemia and two patients had diabetes. Among controls, three had hypertension, one had hypertension and hyperlipidemia and one had diabetes. The scores of SGRQ revealed a reduction in HRQoL to all domains (Supplementary Table S1) in FILD cases when compared to reference values.

**Table SA1-** Health related quality of life according to St. George's Respiratory Questionnaire in fibrotic interstitial lung disease (FILD) cases.

| **Variables** | **FILD cases (n=27)** | **Reference Values *** |
| --- | --- | --- |
| **Respiratory symptoms** | 40.0 (22.8 – 56.1) | 4.2 (0 – 12.2) |
| **Activity** | 60.6 (35.5 – 72.8) | 0.0 (0.0 – 11.2) |
| **Psychosocial impact** | 36.2 (15.1 – 53.9) | 0.0 (0.0 – 0.0) |
| **Total** | 39.5 (24.4 – 61.0) | 2.7 (0.0 – 6.6) |

FILD cases’ values and reference values are expressed as median (25^th^-75^th^ interquartile range)**. Higher scores indicate worse HQOL.**

***** Median (25^th^-75^th^ interquartile range) for St. George's Respiratory Questionnaire in normal male with no history of respiratory disease, derived from Eur Respir J 2002;19: 405-413).

FILD cases walked less than predicted and presented peripheral oxygen desaturation, increased heart rate, dyspnea and leg fatigue at the end of the 6MWT (Supplementary Table S2).

**Table SA2-** Results of six-minute walk test in the fibrotic interstitial lung disease (FILD) cases.

| ***Variable*** | ***FILD cases (n = 30)*** |
| --- | --- |
| Walked distance (meters) | 510 (414 - 556) |
| Walked distance (% predicted) | 84 (77 - 96) |
| Initial SpO_2_ (%) | 95 (93 - 96) |
| Final SpO_2_ (%) | 86 (72 - 93) |
| Initial heart rate (ppm) | 84 (70 - 97) |
| Final heart rate (ppm) | 118 (104 - 138) |
| Initial Borg dyspnea scale | 0 (0 - 0.5) |
| Final Borg dyspnea scale | 6 (5 - 8) |
| Initial Borg leg fatigue scale | 0 (0 - 0) |
| Final Borg leg fatigue scale | 4 (1 - 7) |

Values expressed as median (25^th^-75^th^ interquartile range). SpO2 = peripheral oxygen saturation; ppm = pulse per minute

Diaphragmatic mobility during QB was similar between healthy controls and FILD cases. However, during DB, diaphragmatic mobility was lower in the FILD cases when compared to healthy controls. During QB, at FRC, the diaphragm of FILD cases was significantly thicker than the healthy controls. But, after a maximal DB, at TLC, the diaphragm of FILD cases was significantly thinner than the healthy controls, resulting in a lower TF in the FILD cases (supplementary Figure S1).

**Figure A1 title *- Additional file - Figure A1***

**Figure S1 legend:** Line graph depicting diaphragmatic mobility at quiet (QB) and deep breathing (DB) and diaphragmatic thickness at functional (FRC) and total lung capacity (TLC) of all subjects (healthy controls and FILD cases) (and another side-by-side line graph of the 30 FILD cases)**.**

Resting dyspnea, exercise tolerance (desaturation and dyspnea at the end of the 6MWT), HRQoL (mainly with the activity and total domains of the SGRQ) and lung function (FVC, FEV_1,_ TLC and DLCO) were correlated with diaphragmatic mobility, thickness and TF measured during DB, but not during QB (supplementary Figure S2 and Figure S3).

**Figure S2 title *- Additional file - Figure A2***

**Figure S2 legend:** Correlation of diaphragmatic mobility during deep breathing and healthy-related quality of life (SGRQ), basal dyspnea (MRC), lung function (FVC) and exercise tolerance during the six-minute walk test among the fibrotic interstitial lung disease (FILD) cases

**Figure S3 title *- Additional file - Figure A3***

**Figure S3 legend:** Correlation of diaphragmatic thickening fraction with quality of life (SGRQ), basal dyspnea (MRC), lung function (FVC) and exercise tolerance (desaturation and dyspnea at the end of the six-minute walk test) in fibrotic interstitial lung disease (FILD) cases.
