# Supplementary material for: Diaphragmatic ultrasound findings correlate with dyspnea, exercise tolerance, health-related quality of life and lung function in patients with fibrotic interstitial lung disease
Source: BMC Pulm Med. 2019 Oct 21;19:183. doi: 10.1186/s12890-019-0936-1 (PMC6802109; doi:10.1186/s12890-019-0936-1)
Supplement: Supplementary file 2 — Additional file 2: Line graph depicting diaphragmatic mobility at quiet (QB) and deep breathing (DB) and diaphragmatic thickness at functional (FRC) and total lung capacity (TLC) of all subjects (healthy controls and FILD cases). Figure S1. Comparison of diaphragmatic mobility between healthy controls and FILD cases. Diaphragmatic mobility during QB was similar between groups (p = 0.95), but during DB, diaphragmatic mobility was lower in the FILD cases when compared to healthy controls (p < 0.01). At FRC, the diaphragm of FILD cases was significantly thicker (p 0.01) than the healthy controls. But, at TLC, the diaphragm of FILD cases was significantly thinner than the healthy controls (p < 0.01). [file 12890_2019_936_MOESM2_ESM.docx]

**Diaphragmatic ultrasound findings correlate with dyspnea, exercise tolerance, health-related quality of life and lung function in patients with fibrotic interstitial lung disease.**

**Pauliane Vieira Santana PhD ^1^**

**Leticia Zumpano Cardenas, RT, PhD^1,2^**

**André Luis Pereira de Albuquerque, MD, PhD ^1,3^**

**Carlos Roberto Ribeiro de Carvalho, MD, PhD ^1^**

**Pedro Caruso, MD, PhD ^1,2^**

^1^ Pulmonary Division, Heart Institute (InCor), Hospital das Clínicas, Faculdade de Medicina da Universidade de São Paulo, São Paulo, Brazil

^2^ Intensive Care Unit, AC Camargo Cancer Center, São Paulo, Brazil

^3^ Hospital Sírio Libanês, São Paulo, Brazil
