# Supplementary material for: Diaphragmatic ultrasound findings correlate with dyspnea, exercise tolerance, health-related quality of life and lung function in patients with fibrotic interstitial lung disease
Source: BMC Pulm Med. 2019 Oct 21;19:183. doi: 10.1186/s12890-019-0936-1 (PMC6802109; doi:10.1186/s12890-019-0936-1)
Supplement: Supplementary file 4 [file 12890_2019_936_MOESM4_ESM.docx]

r = -0.38

r = -0.44

r = -0.54

r = 0.68

r = -0.41

r = -0.50
